# Using information theory to identify redundancy in common laboratory tests in the intensive care unit

**DOI:** 10.1186/s12911-015-0187-x

**Published:** 2015-07-31

**Authors:** Joon Lee, David M. Maslove

**Affiliations:** 1 School of Public Health and Health Systems, University of Waterloo, Waterloo, Canada; 2 Department of Medicine & Critical Care Program, Queen’s University, Kingston, Canada

## Abstract

**Background:**

Clinical workflow is infused with large quantities of data, particularly in areas with enhanced monitoring such as the Intensive Care Unit (ICU). Information theory can quantify the expected amounts of total and redundant information contained in a given clinical data type, and as such has the potential to inform clinicians on how to manage the vast volumes of data they are required to analyze in their daily practice. The objective of this proof-of-concept study was to quantify the amounts of redundant information associated with common ICU lab tests.

**Methods:**

We analyzed the information content of 11 laboratory test results from 29,149 adult ICU admissions in the MIMIC II database. Information theory was applied to quantify the expected amount of redundant information both between lab values from the same ICU day, and between consecutive ICU days.

**Results:**

Most lab values showed a decreasing trend over time in the expected amount of novel information they contained. Platelet, blood urea nitrogen (BUN), and creatinine measurements exhibited the most amount of redundant information on days 2 and 3 compared to the previous day. The creatinine-BUN and sodium-chloride pairs had the most redundancy.

**Conclusions:**

Information theory can help identify and discourage unnecessary testing and bloodwork, and can in general be a useful data analytic technique for many medical specialties that deal with information overload.

## Background

Modern medical practice is increasingly a data-driven enterprise. Conventional data based on clinical observation and blood tests are collected in large quantities, with newer modalities such as high-resolution imaging studies and genome-wide assays proliferating rapidly. Strategies are needed both to limit the use of unhelpful tests, and to identify from among the resulting data those that are relevant, and those that are merely distractions.

In the Intensive Care Unit (ICU), likely the most data-rich environment in the hospital, enhanced monitoring and frequent testing are common. Repeated bloodwork can lead to patient harm in a number of ways. First, frequent phelobotomy can cause anemia, and increase the need for blood transfusions. In one observational study in the ICU, patients were phlebotomized between 40 and 70 mL per day, for an average total volume of more than 1 L over the course of their stay [1]. While conservative blood sampling strategies have been shown to reduce daily phlebotomy volumes to as little as 8 mL per day [2], such strategies are not widely used.

Second, frequent blood draws can cause patient discomfort, especially for those patients who do not have indwelling catheters and who therefore require venipuncture for sample collection. Blood samples are often collected very early in the morning, which can disrupt sleep and lead to delirium. Lastly, increased frequency of testing enhances the risk of false positive results, and may increase the risk that clinically irrelevant findings lead to changes in management. We know of no objective approach to determining which blood tests are most informative on a system-wide level. As such, there is a need to develop strategies for the rational use of blood tests both in the ICU, and beyond.

One approach to refining testing strategies, popularized by the Choosing Wisely campaign [3], has been to discourage clinicians and patients from undertaking tests that are unnecessary or unhelpful. A laboratory test utilization management toolbox has also been developed to guide clinicians on appropriate utilization of needed lab tests [4]. In addition, Cismondi et al. have applied artificial intelligence to study the predictability of future lab test results in a patient cohort with gastrointestinal bleeding, extracted from the same public ICU database that the present study analyzed [5].

In this study, we explored an alternative approach in which the science of information theory is used to identifying areas of overlap or redundancy between clinical tests. Information theory has long been used in medical research in numerous domains including neuroscience [6], molecular biology [7], cell signaling [8], genomics [9], and medical imaging [10]. In the area of medical decision making, information theory has been applied to the evaluation of diagnostic tests, including laboratory tests [11–13]. In these formulations, the quantity of information gained by performing a test is related to the difference in the uncertainty in outcome (i.e., entropy) associated with the probability distribution of the diagnosis both prior to and following the test.

Information theory provides a fundamental framework for quantifying information [14]. The central idea is that the amount of information contained in a variable is related to its randomness. For example, the tossing of a biased coin with 90 % chance of turning up heads contains less information than a fair coin toss because the result is more predictable. In general, less randomness means more predictability, and vice versa.

We used constructs from information theory, namely entropy, conditional entropy, and mutual information, to analyze lab test results from a large ICU database. Entropy, conditional entropy, and mutual information are fundamental metrics that comprise information theory, and can, by definition, describe the information contents of a single variable as well as of the association between two variables. We hypothesized that in addition to quantifying the relationship between diagnostic tests and disease states, information theory could be useful in addressing clinical information overload by identifying redundancies between laboratory tests done concurrently, as well as those done sequentially for the purpose of daily monitoring. We further hypothesized that specific pairs of laboratory values with shared physiologic mechanisms would exhibit greater redundancy than pairs that do not.

The objective of this proof-of-concept study was to employ information theory to quantify the amount of information contained in common laboratory tests, the extent of redundancy between consecutive days of sampling, and the redundancy associated with pre-specified pairs of ICU lab tests. These pairs included creatinine and blood urea nitrogen (BUN) (both related to renal function), bicarbonate and lactate (related physiological because the former acts as a buffer for the latter), sodium and chloride (both constituents of a common resuscitation fluid), and white blood cell count (WBC) and platelet count (hypothesized to contain less redundancy).

## Methods

We extracted laboratory test results from the MIMIC II database that contains data from over 25,000 adult ICU patients [15]. The ICU admissions in MIMIC II typically have 1–4 lab measurements per day. The following lab tests were investigated: hematocrit, platelet count, white blood cell count (WBC), glucose, HCO_3_, potassium, sodium, chloride, BUN, creatinine, and lactate. These variables were chosen for their pervasiveness in and high relevance to intensive care. A total of 29,149 adult ICU admissions were analyzed and missing data were excluded. Because MIMIC II is a fully de-identified public database, the need for informed consent and research ethics review was waived. MIMIC II data were extracted in Oracle SQL Developer (version 3.2.09).

**Fig. 1:**
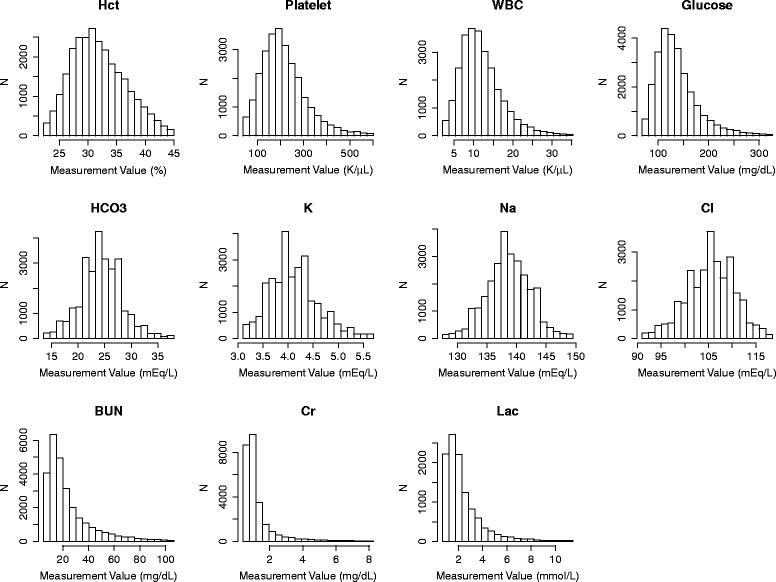
Histograms of lab test values from ICU day 1. *Hct* hematocrit, *WBC* white blood cell count, *K* potassium, *Na* sodium, *Cl* chloride, *BUN* blood urea nitrogen, *Cr* creatinine, *Lac* lactate

For each ICU admission, the median value of each lab test from each ICU day was used for analysis. For each variable, values less than the 1st percentile and greater than the 99th percentile were regarded as outliers and discarded. The use of median instead of mean as well as the 1st and 99th percentile cutoffs reduced the effects of outliers which commonly exist in raw clinical data such as MIMIC II due to recording and measurement errors. Also, median was particular appropriate since most lab tests exhibited skewed distributions as shown in Fig. 1. Following the exclusion of outliers, the remaining values were discretized into 20 bins of equal width. The choice of 20 bins was informed by visual inspection of the distributions of the lab test values to ensure adequate bin size; Fig. 1 shows the histogram of each lab test using 20 bins. The expected amount of information that each lab test (X) contains, known as the *entropy* of X and represented as H(X), was calculated as:

$$ H(X)=-{\displaystyle \sum_i}P\left({x}_i\right){ \log}_2P\left({x}_i\right) $$

where P(x_i_) is the probability of X resulting in the possible value x_i_ and the summation enumerates all possible values of X. When the logarithm with base 2 is used as above, H(X) is expressed in *bits* as a unit of measurement. The maximum attainable entropy (i.e., when all 20 bins are equally likely) in each lab test was therefore 20∗(1/20)∗[−log_2_(1/20)] = 4.32 bits.

We also calculated the expected amount of redundant information between two lab tests X and Y, termed *mutual information* and denoted as I(X;Y), as follows:

$$ I\left(X;Y\right)={\displaystyle \sum_j}{\displaystyle \sum_i}P\left({x}_i,{y}_j\right){ \log}_2\left(\frac{P\left({x}_i,{y}_j\right)}{P\left({x}_i\right)P\left({y}_j\right)}\right) $$

where P(x_i_,y_j_) is the joint probability of X and Y resulting in values x_i_ and y_j_, respectively.

Lastly, we quantified the expected amount of novel information in X after knowing the value of Y, known as *conditional entropy* and denoted as H(X|Y), as follows:

$$ H\left(X\Big|Y\right)=H(X)-I\left(X;Y\right) $$

**Fig. 2:**
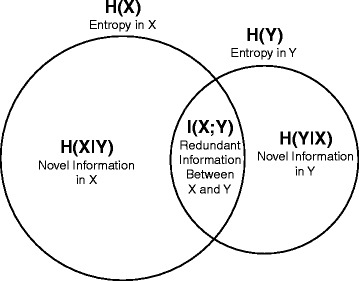
An illustration of information theory applied to two variables. This Venn diagram illustrates: the entropies in the two variables X and Y, represented as H(X) and H(Y), respectively; the mutual information (i.e., redundant information) between X and Y, represented as I(X;Y); and the expected amounts of novel information in X and Y, represented as H(X|Y) and H(Y|X), respectively. H(X) is greater than H(Y), which signifies that X is associated with more randomness and less predictability than Y. H(X|Y) represents the expected amount of novel information left in X when Y is known, and vice versa for H(Y|X)

Figure 2 visually illustrates H(X), H(Y), I(X;Y), H(X|Y), and H(Y|X). Interested readers are directed to additional references for further details on information theory [16, 17].

We first examined the mutual information between measurements from the first three consecutive days in the ICU. We next analyzed the following pairs of variables for their redundant information on each ICU day: creatinine-BUN, HCO_3_-lactate, sodium-chloride, and platelet-WBC. While all final results were from an ICU-wide analysis, the coupling information required in the mutual information calculation, i.e., the joint probability P(x_i_,y_j_), was computed at the individual patient level. Analyses were conducted in R (version 3.1.1).

## Results

Table 1 summarizes the descriptive statistics of the analyzed lab tests, stratified by ICU days. Although almost all ICU admissions had at least one measurement of each lab test on day 1 (except lactate), progressively fewer ICU admissions with lab test results were available in MIMIC II on days 2 and 3. This is expected due to discharged patients, and changes in patient acuity. Across all three ICU days, lactate was the least frequently tested lab variable.

**Table 1:** Descriptive statistics of lab tests

	ICU day 1		ICU day 2		ICU day 3	
	*N*	Mean [SD]	*N*	Mean [SD]	*N*	Mean [SD]
Hematocrit (%)	27,549	31.9 [5.0]	19,307	30.7 [4.4]	12,913	30.4 [4.2]
Platelet (K/μL)	27,347	218.4 [112.2]	18,710	198.8 [110.4]	12,597	196.0 [114.9]
White Blood Cell Count (K/μL)	27,187	12.3 [8.8]	18,675	12.0 [7.6]	12,579	11.8 [7.9]
Glucose (mg/dL)	27,638	139.3 [50.5]	19,167	128.4 [44.2]	12,840	129.1 [43.0]
HCO_3_ (mEq/L)	27,813	24.4 [4.6]	19,077	25.0 [4.6]	12,831	25.5 [4.8]
Potassium (mEq/L)	27,792	4.1 [0.5]	19,475	4.1 [0.5]	13,048	4.0 [0.5]
Sodium (mEq/L)	27,620	138.6 [4.4]	18,881	138.7 [4.5]	12,745	138.9 [4.7]
Chloride (mEq/L)	27,352	105.5 [5.7]	18,760	105.3 [5.6]	12,647	105.0 [5.8]
Blood Urea Nitrogen (mg/dL)	27,599	25.3 [20.9]	18,867	26.2 [21.2]	12,712	28.0 [22.1]
Creatinine (mg/dL)	27,612	1.4 [1.5]	18,883	1.4 [1.5]	12,716	1.5 [1.5]
Lactate (mmol/L)	11,423	2.5 [2.0]	4533	2.2 [2.3]	2861	2.1 [2.2]

The number of adult ICU admissions with at least one measurement (N), as well as the mean and standard deviation (SD) of daily median values from individual ICU admissions are shown for the first 3 days in the ICU

**Fig. 3:**
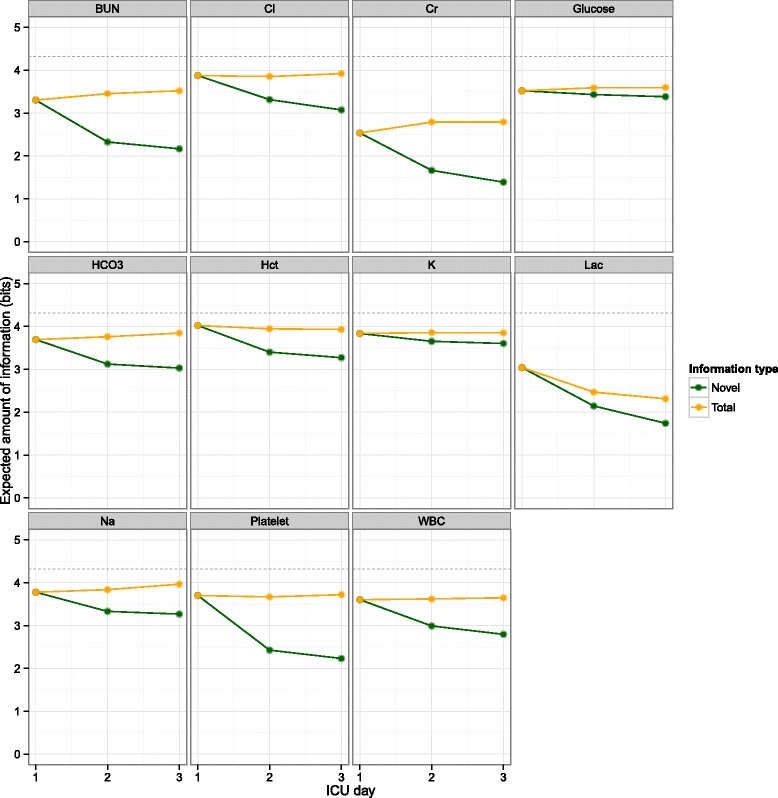
Information contained in daily ICU lab test results. The total and novel amounts of information are plotted as a function of ICU day. The *horizontal dashed lines* at 4.32 bits represent the maximum achievable entropy. The redundant portions (i.e., total minus novel) of day 2 and 3 are the mutual information between days 1 and 2 and between days 2 and 3, respectively. Although all lab tests contain redundant information on days 2 and 3 compared to the previous day, platelet, BUN, and Cr have the least novel information on days 2 and 3. *Hct* hematocrit, *WBC* white blood cell count, *K* potassium, *Na* sodium, *Cl* chloride, *BUN* blood urea nitrogen, *Cr* creatinine, *Lac* lactate

Figure 3 depicts the entropy of each lab test on each ICU day, as well as how much of the day 2 and 3 information was redundant compared to the previous day. Creatinine was the most predictable variable. Across ICU days, most variables were stable in terms of entropy but resulted in decreasing amounts of novel information. Platelet count, BUN, and creatinine were the most redundant variables on days 2 and 3 given their values on days 1 and 2, respectively.

The decreasing entropy in lactate was likely due to the increasing number of normal lactate values (i.e., less random) over time. As shown in Table 1, the daily mean lactate values and corresponding standard deviations (in square brackets) were (in mmol/L): day 1: 2.5 [2.0]; day 2: 2.2 [2.3]; day 3: 2.1 [2.2].

**Fig. 4:**
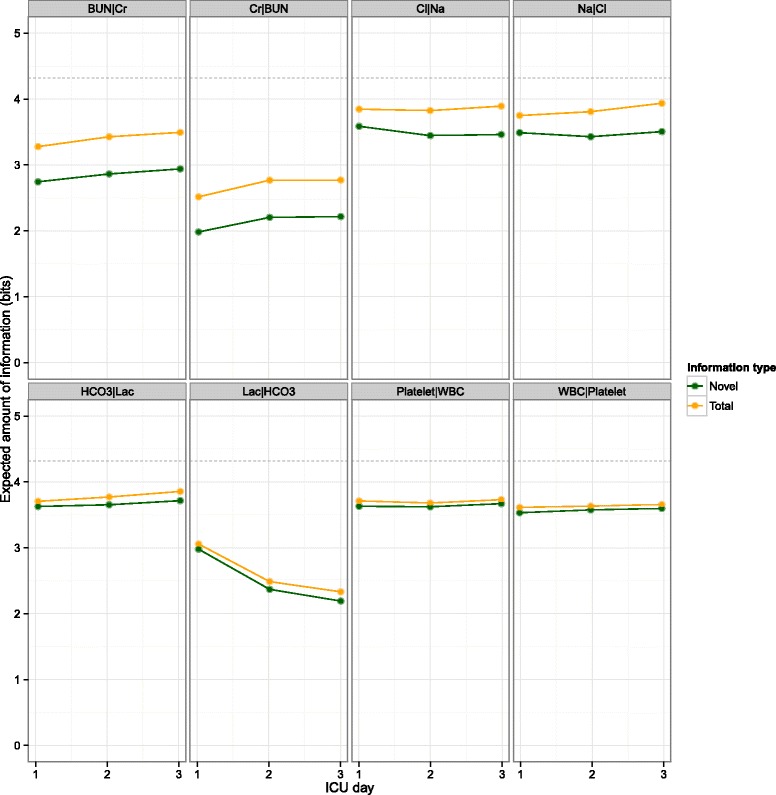
Redundant information in selected ICU lab test pairs. Information contents are plotted as a function of ICU day. The *horizontal dashed lines* at 4.32 bits represent the maximum achievable entropy. Each lab test pair is shown asymmetrically side by side; e.g., BUN|Cr and Cr|BUN refer to the information in BUN given creatinine and that in creatinine given BUN, respectively. Because Cr has a smaller entropy than BUN, the redundant information between the two variables represents a greater percentage of the entropy in Cr than that in BUN. *WBC* white blood cell count, *Na* sodium, *Cl* chloride, *BUN* blood urea nitrogen, *Cr* creatinine, *Lac* lactate

In the pairwise analysis, the creatinine-BUN and sodium-chloride pairs contained more redundant information than others (Fig. 4). While the absolute amount of redundant information is identical to both variables in the pair, it represents different relative portions with respect to the entropy in each variable. For example, BUN reduces the unpredictability in creatinine more than creatinine does for BUN, relatively speaking.

## Discussion

Intensive care clinicians face a deluge of data on morning rounds, which are typically processed heuristically based on previous experience. Our findings provide objective confirmation of what heuristic methods devise implicitly, namely that the values of some lab tests are more easily predicted than others, and that the information gain between subsequent ICU days is greater for some tests than others. Specifically, we found that the creatinine-BUN pair, the bicarbonate-lactate pair, and the sodium-chloride pair contained a greater degree of redundancy than the WBC-platelet pair. These relationships likely reflect shared physiologic mechanisms, and suggest ways in which policies on lab testing might be developed to capitalize on redundancy, and avoid unnecessary testing.

Our findings have direct implications for medical decision making in the ICU, specifically related to the ordering of daily bloodwork. The ordering of routine bloodwork is pervasive in the ICU [18]. While estimates of inappropriate testing in general may vary, one view is that a significant proportion of bloodwork done in the ICU setting is unnecessary [19]. A number of strategies have been implemented to limit unnecessary bloodwork, including education programs, changes in ordering protocols, clinical decision supports, quota systems, and cost transparency [18]. To our knowledge, ours is the first study to quantify the redundant information content between various blood tests. We believe our results provide an empirical basis upon which rational blood test ordering strategies can be developed.

The redundancy in information between BUN and creatinine mentioned above suggests that if one is known, the other can be inferred with reasonable confidence. If a hospital were to restrict one of these tests, our results suggest it may be better to reduce testing of creatinine, and to order BUN alone instead, reflecting the asymmetrical relationship between these two tests. While the addition of creatinine to BUN values may not on average add much novel information, clinical judgment is still needed to assess the value added from this test on a case-by-case basis (such as when upper gastrointestinal bleeding is suspected, in which case the BUN data may provide information beyond its reflection of renal function) [20]. Similarly, while the platelet count on day 2 of the ICU admission may on average contain little novel information in light of the day 1 value, ongoing measurement of platelet count may be warranted if the day 1 value was abnormal, if the value was changing significantly from day to day, or if the value had to be known precisely for the purpose of planning invasive procedures.

Our study has limitations that merit further discussion. First, our main claim on unnecessary testing is based on the theoretical predictability of lab tests with respect to joint probability distributions. The actual achievable predictive performance should be investigated further in a follow-up study. Second, with respect to the clinical applicability of our findings, we note that our results apply to the information content of laboratory measurements, rather than the biological processes they represent. As such, limited conclusions can be drawn about the natural history of critical illness from a physiologic standpoint. Our results do, however, have bearing on common practices in the ICU related to the frequency of laboratory testing. Frequent blood draws remain commonplace in most ICUs, especially within the first few days of admission to ICU. Our results suggest that even in these periods during which physiologic changes may be most pronounced, the amount of novel information gleaned from repeated blood testing may be limited. Third, the present study did not incorporate other clinical factors that might influence the information contents in lab tests (e.g., clinical outcomes might be associated with the predictability of certain lab tests). Lastly, as a retrospective data analysis, our findings should not be used to support immediate widespread changes in laboratory practices, but rather lend support to the notion that over-testing may be occurring and warrants further study.

Prior applications of information theory to medical decision making have largely focused on the evaluation of diagnostic tests, by determining the conditional probability of a disease state given the result of the test. Using similar methods, we defined common information theory parameters including entropy and mutual information for common lab tests done concurrently or sequentially, without reference to a final diagnosis. Our findings suggest that this approach may have utility in analyzing the increasing abundance of biomedical Big Data. Future work will focus on adapting these methods to developing strategies to limit excessive testing in the ICU, and measuring redundancy among three or more lab tests.

## Conclusions

Information theory can be a useful tool in objectively quantifying the amount of redundancy associated with lab tests. As this proof-of-concept study showed, information theory can help clinicians decide which lab tests are truly needed.

## Availability of supporting data

Although MIMIC II is a public database, gaining access to MIMIC II requires completing human subjects training and signing a data use agreement (visit http://physionet.org/mimic2 for instructions). The clinical part of MIMIC II is a relational database and interested researchers are free to write their own SQL queries to extract data from it after becoming an approved MIMIC II user. However, for those who are interested in replicating or extending our particular research results, we are able to share our SQL query that was used to extract the raw data for this study from MIMIC II.
